# Intrapartum epidural analgesia and breastfeeding: a prospective cohort study

**DOI:** 10.1186/1746-4358-1-24

**Published:** 2006-12-11

**Authors:** Siranda Torvaldsen, Christine L Roberts, Judy M Simpson, Jane F Thompson, David A Ellwood

**Affiliations:** 1 NSW Centre for Overweight and Obesity, Level 2, K25 Medical Foundation Building, The University of Sydney, NSW 2006, Australia; 2 Centre for Perinatal Health Services Research, Building DO2, The University of Sydney, NSW 2006, Australia; 3 School of Public Health, The University of Sydney, NSW 2006, Australia; 4 Women's & Children's Hospitals Australasia, Level 1, 99 Northbourne Ave, Turner ACT 2612, Australia; 5 The Australian National University Medical School, The Canberra Hospital, ACT 2606, Australia

## Abstract

**Background:**

Anecdotal reports suggest that the addition of fentanyl (an opioid) to epidural analgesia for women during childbirth results in difficulty establishing breastfeeding. The aim of this paper is to determine any association between epidural analgesia and 1) breastfeeding in the first week postpartum and 2) breastfeeding cessation during the first 24 weeks postpartum.

**Methods:**

A prospective cohort study of 1280 women aged ≥ 16 years, who gave birth to a single live infant in the Australian Capital Territory in 1997 was conducted. Women completed questionnaires at weeks 1, 8, 16 and 24 postpartum. Breastfeeding information was collected in each of the four surveys and women were categorised as either fully breastfeeding, partially breastfeeding or not breastfeeding at all. Women who had stopped breastfeeding since the previous survey were asked when they stopped.

**Results:**

In the first week postpartum, 93% of women were either fully or partially breastfeeding their baby and 60% were continuing to breastfeed at 24 weeks. Intrapartum analgesia and type of birth were associated with partial breastfeeding and breastfeeding difficulties in the first postpartum week (p < 0.0001). Analgesia, maternal age and education were associated with breastfeeding cessation in the first 24 weeks (p < 0.0001), with women who had epidurals being more likely to stop breastfeeding than women who used non-pharmacological methods of pain relief (adjusted hazard ratio 2.02, 95% CI 1.53, 2.67).

**Conclusion:**

Women in this cohort who had epidurals were less likely to fully breastfeed their infant in the few days after birth and more likely to stop breastfeeding in the first 24 weeks. Although this relationship may not be causal, it is important that women at higher risk of breastfeeding cessation are provided with adequate breastfeeding assistance and support.

## Background

Breastfeeding difficulties in the early postpartum period, especially if combined with inadequate support and advice, may lead new mothers to give up breastfeeding. Many factors influence the establishment of breastfeeding, including the type of labour and birth [1-4]. Local anecdotal reports were suggesting that the addition of fentanyl (an opioid) to epidural analgesia was resulting in difficulty establishing breastfeeding. Whilst the effects of epidurals on some obstetric consequences, including longer labour and an increased risk of an instrumental delivery [5], are well established, the impact, if any, of epidurals on breastfeeding is less clear.

A study conducted in Western Australia of 484 primiparous women with spontaneous vaginal births found epidural analgesia was associated with shorter breastfeeding duration (adjusted hazard ratio 1.44, 95% CI 1.04, 1.99) [4]. Similarly, a study undertaken in Lapland of 64 primiparous women with spontaneous vaginal births found women who had epidural analgesia were more likely to be either partially breastfeeding or formula feeding at 12 weeks postpartum than women who had not had epidural analgesia (relative risk 2.27, 95% CI 1.27, 4.04) [6]. Three of four small (56 ≤ n ≤ 189) North American studies examining the effects of epidurals on breastfeeding initiation and duration found no association [7-9], in spite of one study demonstrating an association between epidurals and reduced neonatal suckling scores [9]. The fourth study (n = 108) found reduced rates of breastfeeding at six months among privately insured women with an epidural (30% vs 50%) [10].

This study aimed to determine whether there is any association between intrapartum epidural analgesia/anaesthesia and 1) breastfeeding in the first week postpartum and 2) breastfeeding cessation during the first 24 weeks postpartum.

## Methods

This population-based, prospective cohort study has been described in detail previously [11]. In brief, participants were women resident in the Australian Capital Territory (ACT) aged 16 years and over, who gave birth to a live baby between March and October 1997 in any of the ACT's two public hospitals (one included a birth centre), two private hospitals, or at home. Women were excluded if their baby was admitted to the neonatal intensive care unit or adopted, if critically ill themselves (i.e. too ill to be able to be asked to participate/give informed consent such as if they were admitted to the intensive care or psychiatric units or had an extended stay in delivery suite for medical reasons) or unable to give informed consent or complete the questionnaires for other reasons. For the analyses presented in this paper, women with multiple pregnancies were also excluded.

The study was approved by the ACT Department of Health & Community Care Research Ethics Committee, and the ethics committees of participating hospitals. Postnatal ward or domiciliary midwives gave information sheets to women in the first few days after giving birth. Women gave written informed consent when completing the first questionnaire as close to day four as possible (88% of respondents had completed the questionnaire by day 6). Women were then mailed questionnaires at 8, 16, and 24 weeks postpartum. The first questionnaire included sociodemographic characteristics of the mother and her partner, obstetric details and information on breastfeeding. Breastfeeding information was collected in each of the four surveys and women were categorised into one of three levels – fully breastfeeding (includes expressed breast milk), partially breastfeeding (breast milk plus some formula) or not breastfeeding at all. If women had stopped breastfeeding since the previous survey, they were asked when they stopped breastfeeding, to the nearest two weeks.

For each study participant, type of labour analgesia was classified into one of the following five mutually exclusive categories: 1) non-pharmacological only (natural methods such as breathing exercises, massage, moving about, hypnosis); 2) gas (nitrous oxide, no other pharmacological agent); 3) pethidine (+/- gas); 4) epidural (analgesia or anaesthesia, +/- pethidine +/- gas); or 5) general anaesthetic (+/- any of 1–4). The epidural solution in use at the time was bupivacaine 0.16% with fentanyl 3.3 μg/ml. This was given as patient controlled epidural analgesia with a 4–6 ml bolus and a 15 minute lockout (Dr Cliff Peady, Acting Director Anaesthetics, The Canberra Hospital, personal communication).

### Data analysis

Associations between demographic and intrapartum factors and breastfeeding in the first week postpartum were investigated using χ^2 ^tests. Odds ratios (OR) were estimated using logistic regression models to determine predictors of partial and no breastfeeding in the first week. For all models, variables that were individually significant at p < 0.05 were included initially; the variables with the highest p-value were then removed one at a time until only those with p < 0.05 remained. Some of the variables predictive of partial breastfeeding were on the causal pathway of others and so could not be included in a multivariate model. For example if there is no labour then the type of birth is caesarean section and the only analgesia options are epidural or general anaesthetic. Kaplan-Meier survival probability estimates were used to summarise time to cessation of breastfeeding during the first 24 weeks postpartum, among women who were partially or fully breastfeeding in the first week after birth. The hazard ratios (HR) of breastfeeding cessation in the first 24 weeks and their 95% confidence intervals (CI) of each exposure level were estimated by fitting a Cox proportional hazards regression model. All data analyses were undertaken using SAS version 8.2 (SAS Institute, Cary, NC).

## Results

Of 1961 ACT residents asked to participate in the study, 105 were ineligible and 1295 (70%) of the remainder consented to participate. Compared with all women who gave birth in 1997, study participants were slightly older, more likely to be married or in a defacto relationship and to have given birth in a private hospital [11]. More detail about respondents and the women who were lost to follow-up can be found in the original publication of the cohort study [11]. Of the 1295 women, 15 (1.2%) gave birth to twins and were excluded from these analyses leaving 1280 women; 1178 (92%) of whom were retained in the study to 24 weeks. Of the 1260 women on whom data were available, 416 (33%) had epidurals. All women who gave birth vaginally with epidural analgesia also used pethidine. Maternal age, parity, type of birth and onset of labour were significantly associated with type of intrapartum analgesia but maternal education was not (Table 1).

**Table 1:** Method of analgesia by demographic and obstetric factors

	**Non-pharmacological** **n (%)**	**Gas** **n (%)**	**Pethidine** **n (%)**	**Epidural** **n (%)**	**General anaesthetic** **n (%)**	**p-value from χ^**2 **^test**
	**n = 312**	**n = 190**	**n = 292**	**n = 416**	**n = 50**	
***Maternal age***						0.002
16–19	5 (2)	4 (2)	9 (3)	8 (2)	2 (4)	
20–24	28 (9)	17 (9)	54 (18)	45 (11)	3 (6)	
25–29	99 (32)	63 (33)	99 (34)	153 (37)	11 (22)	
30+	179 (58)	106 (56)	130 (45)	210 (50)	34 (68)	
***Maternal education***						0.32
Up to year 11	61 (20)	43 (23)	63 (22)	74 (18)	10 (20)	
Year 12	70 (23)	43 (23)	80 (27)	98 (24)	5 (10)	
Trade/certificate/diploma	61 (20)	31 (16)	57 (20)	87 (21)	11 (22)	
Degree	118 (38)	73 (38)	92 (32)	155 (37)	24 (48)	
***Type of birth***						<0.0001
Vaginal	306 (98)	183 (96)	262 (90)	116 (28)	0	
Instrumental	6 (2)	7 (4)	30 (10)	128 (31)	0	
CS with labour	0	0	0	64 (15)	19 (38)	
CS no labour	0	0	0	108 (26)	31 (62)	
***Onset of labour***						<0.0001
Spontaneous	258 (83)	148 (78)	201 (69)	162 (39)	11 (22)	
Induced	53 (17)	41 (22)	89 (31)	144 (35)	8 (16)	
No labour	0	0	0	108 (26)	31 (62)	
***Parity***						<0.0001
No previous births	84 (27)	62 (33)	157 (54)	225 (54)	19 (38)	
≥1 previous births	227 (73)	128 (67)	134 (46)	191 (46)	31 (62)	
***Length of labour***^***1***^						<0.0001
<2 hours	52 (17)	24 (13)	12 (4)	6 (2)	1 (5)	
2–12 hours	224 (72)	144 (76)	200 (68)	142 (47)	11 (58)	
12–24 hours	32 (10)	16 (8)	67 (23)	105 (34)	4 (21)	
>24 hours	3 (1)	5 (3)	13 (5)	51 (17)	3 (16)	

^1^among women who laboured

**Figure 1:**
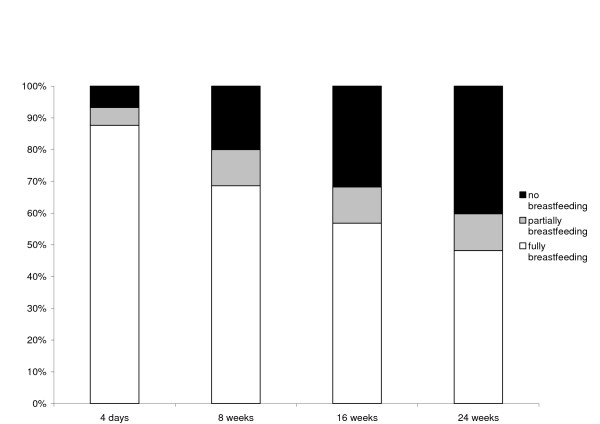
Breastfeeding at 4 days, 8 weeks, 16 weeks and 24 weeks postpartum.

**Figure 2:**
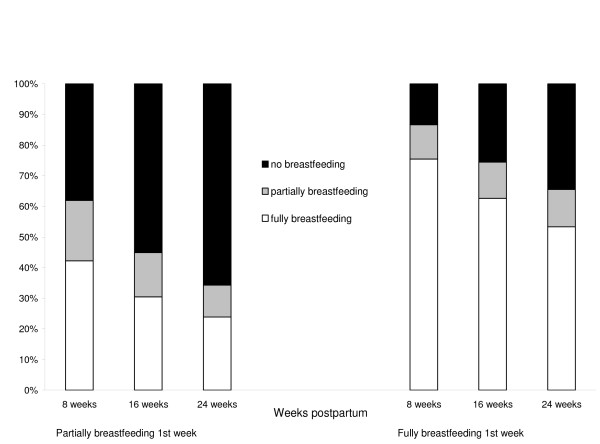
Breastfeeding status at 8, 16 and 24 weeks postpartum by breastfeeding status in the first postpartum week.

In the first week postpartum, 1182 (93%) women on whom breastfeeding data were available were either fully or partially breastfeeding their baby, and 704 (60%) of these women were continuing to breastfeed at 24 weeks (Figure 1). Of the 85 women who were not breastfeeding at all in the first week, 41 (48%) indicated that this was what they had planned. Compared with fully breastfeeding, predictors of partial breastfeeding differed from predictors of no breastfeeding. No intrapartum factors were associated with no breastfeeding; the only factor associated with no breastfeeding was maternal education (p < 0.0001), women with less education being much more likely not to breastfeed at all in the first week after birth (among women who were not breastfeeding at all, the OR for year 12 education was 3.11, 95% CI 1.42, 6.79, compared with women who had a degree, and the OR for year 11 or less education was 7.63, 95% CI 3.72, 15.56). Three of the four factors significantly associated with partial breastfeeding in the first week were intrapartum factors (Table 2). These were: intrapartum analgesia (p < 0.0001), type of birth (p < 0.0001) and onset of labour (p = 0.0003). Parity was also significant (p = 0.0006). Of the five intrapartum analgesia categories, only epidurals and general anaesthetic were significantly associated with an increased risk of partial breastfeeding in the first week, after adjusting for parity (Table 2). When we restricted the analyses to women who had vaginal births, this association was weaker and, after adjusting for parity, it was no longer statistically significant (OR 2.33, 95% CI 0.92, 5.90). Education was not significant, nor was age, hospital type (private or public) or having a small for gestational age infant, and although length of labour was significant in the univariate analysis, it was no longer significant after adjusting for parity. Women who reported partially breastfeeding in the first week were almost twice as likely to have ceased breastfeeding by 24 weeks compared with women who were fully breastfeeding in the first week (relative risk 1.91, 95% CI 1.57, 2.31, Figure 2). The predictors of breastfeeding difficulties in the first week after birth were similar to the predictors of partial breastfeeding. Among women reporting difficulty breastfeeding in the first week, the OR for epidural, adjusted for parity, was 2.04 (95% CI 1.39 to 3.00). This association remained significant when we restricted the analysis to women who had vaginal births (OR 1.75, 95% CI 1.13, 2.70).

**Table 2:** Predictors of partial breastfeeding in the first postpartum week.

	**Partial breastfeeding** **(compared with fully breastfeeding)** **adjusted OR (95% CI)**
***Analgesia***^**1**^	
Non-pharmacological	1 (referent group)
Gas	1.46 (0.48, 4.45)
Pethidine	0.97 (0.34, 2.75)
Epidural	3.61 (1.57, 8.29)
General anaesthetic	9.25 (3.22, 26.59)
***Parity***^***2***^	
Multiparous	1 (referent group)
Primiparous	2.59 (1.51, 4.45)
***Type of birth***^**1**^	
Vaginal	1 (referent group)
Instrumental	1.82 (0.91, 3.65)
CS* no labour	4.41 (2.25, 8.64)
CS with labour	6.22 (3.15, 12.30)
***Onset of labour***^**1**^	
Spontaneous	1 (referent group)
Induced	1.86 (1.07, 3.22)
No labour	3.73 (1.92, 7.24)

^1^adjusted for parity, ^2^adjusted for analgesia, *CS = Caesarean section

Note: Cannot include analgesia, type of birth and onset of labour in the one model as some variables are on the causal pathway of others

**Figure 3:**
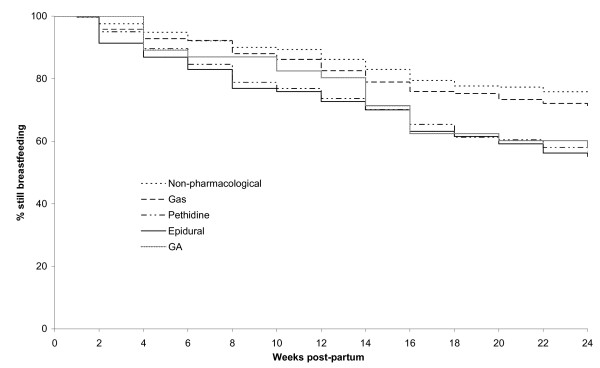
Kaplan-Meier survival curves of the time to cessation of breastfeeding stratified by method of analgesia, among women who were breastfeeding in the first week after birth.

Kaplan-Meier survival curves show a significant difference in breastfeeding cessation rates (i.e. women who were fully or partially breastfeeding in the first week later reporting not breastfeeding at all) by method of intrapartum analgesia (p < 0.0001), with women who used no pharmacological analgesia having the lowest rates of breastfeeding cessation during the first 24 weeks and women who used epidurals having the highest rate (Figure 3). Cox proportional hazards analysis found that, among women who were breastfeeding in the first week, intrapartum analgesia, maternal age and education were all associated with breastfeeding cessation in the first 24 weeks (p < 0.0001), with women who had pethidine or an epidural significantly more likely to stop breastfeeding in the first 24 weeks than women who used non-pharmacological methods of pain relief (Table 3). The few women who had a general anaesthetic for the birth of their baby were not included in the Cox proportional hazard model because, as can be seen from Figure 3, the HR of breastfeeding cessation in this group was not constant over time. Parity and marital status were significant in univariate analyses but not in the multivariate model. The following variables were not significant (p > 0.05) so were not included in the model: type of birth, onset of labour, length of labour, mother's country of birth, maternal employment in the past 12 months, plan to return to work, whether this pregnancy was a result of assisted reproductive technology, gender of the baby, whether the baby was small for gestational age, and measures of maternal social support, resilience and vulnerability. Although type of birth and parity were not significant, we undertook three further analyses where we restricted the model to vaginal births and then primiparous and multiparous women. The survival curves and adjusted hazard ratios were virtually unchanged in these restricted analyses.

**Table 3:** Predictors of breastfeeding cessation in the first 24 weeks^1^

	Crude HR (95% CI)	Adjusted HR^2 ^(95% CI)	n/N (%) breastfeeding at 24 weeks
***Analgesia***			
Non-pharmacological	1 (referent group)	1 (referent group)	210/292 (72)
Gas	1.21 (0.84, 1.74)	1.20 (0.83, 1.74)	112/166 (67)
Pethidine	1.95 (1.45, 2.63)	1.67 (1.23, 2.25)	139/261 (53)
Epidural	2.07 (1.57, 2.72)	2.02 (1.53, 2.67)	206/396 (52)
***Maternal age***			
30+	1 (referent group)	1 (referent group)	446/605 (74)
25–29	2.43 (1.95, 3.03)	2.21 (1.76, 2.78)	183/384 (48)
20–24	3.51 (2.65, 4.65)	2.67 (1.99, 3.58)	45/125 (36)
16–19	4.52 (2.60, 7.84)	3.19 (1.82, 5.61)	4/19 (21)
***Maternal education***			
Degree	1 (referent group)	1 (referent group)	321/424 (76)
Trade/certificate/diploma	1.78 (1.32, 2.39)	1.67 (1.24, 2.26)	132/225 (59)
Year 12	2.39 (1.84, 3.11)	2.01 (1.53, 2.64)	141/275 (51)
Up to year 11	2.97 (2.25, 3.91)	2.45 (1.84, 3.27)	81/205 (40)

^1^excluding women who had a general anaesthetic

^2^analgesia, maternal age and education in the model

To see if all the observed effect in the epidural group could have been due to the pethidine, we also undertook a Cox proportional hazards analysis using re-categorised analgesia groups. Instead of epidural +/- pethidine, we used epidural only and pethidine plus epidural, although it should be noted that *all *women in the epidural only group had a caesarean section. The HR for epidural only (adjusted for age and education) was 2.07 (95% CI 1.44, 2.96) and the HR for pethidine plus epidural was 1.73 (95% CI 1.30, 2.25).

In order to determine whether the increased rate of breastfeeding cessation among women who had an epidural was all due to those who were partially breastfeeding in the first week, we also undertook a Cox proportional hazards analysis of women who were fully breastfeeding in the first week who later reported either partially breastfeeding or not breastfeeding at all. Women who had an epidural during their labour and were fully breastfeeding in the first week were significantly more likely to stop fully breastfeeding than women who did not use any pharmacological agents during their labour (HR 1.59, 95% CI 1.25, 2.02); hence partial breastfeeding in the first week does not completely explain the increased breastfeeding cessation rate.

## Discussion

In this study, women who had epidural analgesia, a general anaesthetic and/or a caesarean section for the birth of their baby were more likely to be partially breastfeeding their baby in the first week postpartum. Partial breastfeeding in the first week may be indicative of difficulty establishing breastfeeding and is important because these women are at increased risk of stopping breastfeeding altogether. Two-thirds of the women who were partially breastfeeding in the first week were not breastfeeding at all by 24 weeks, compared with only one third of women who were fully breastfeeding in the first week. The type and amount of breastfeeding support given to these women is not known but may be an important factor in women continuing to breastfeed in spite of initial difficulties. International recommendations advise that women exclusively breastfeed their infants until six months of age and continue breastfeeding until at least 12 months of age [12].

Surprisingly, there was no overlap of predictors of partial and no breastfeeding in the first week. To the best of our knowledge, this has not been previously reported. The fact that no intrapartum factors were associated with not breastfeeding at all suggests that not breastfeeding has more to do with maternal preferences, which in turn are influenced by education. Women with a low level of education are at increased risk of both not initiating breastfeeding and, if they do breastfeed in the first few days, stopping in the first 24 weeks. Education has been previously reported to be associated with breastfeeding rates [13]. Other previously reported risk factors for breastfeeding cessation, such as planning to return to work, were not significant in this cohort of women.

Intrapartum analgesia, type of birth and onset of labour are inextricably linked and it is hard to evaluate the relative contribution of each on breastfeeding. The effects of analgesia and type of birth are difficult to separate as each of these variables could be on the causal pathway of the other. When the analyses were restricted to women who had vaginal births, the strength of association between epidurals and partial breastfeeding and between epidurals and breastfeeding difficulties was weaker, and the association between epidurals and partial breastfeeding lost statistical significance. This suggests that at least some of the effect of epidurals could be due to its association with type of birth.

Although type of birth and parity were strong predictors of partial breastfeeding in the first week, neither was associated with breastfeeding cessation in the first 24 weeks postpartum. The only variable to be significantly associated with both partial breastfeeding in the first week and breastfeeding cessation during the first 24 weeks was intrapartum analgesia, with women in the epidural group being twice as likely to stop breastfeeding altogether in the first 24 weeks after birth as women who had non-pharmacological methods of analgesia during their labour. However, women stop breastfeeding for a variety of reasons, and this study did not collect information on all possible reasons.

Although none of the recorded potential confounders was significant, some or all of the association could be due to characteristics of the women who choose different sorts of analgesia. Women who choose non-pharmacological methods may be more likely to continue breastfeeding for at least the first 24 weeks and this could explain, either partly or wholly, the association between intrapartum analgesia and breastfeeding cessation. A previous study, in which women consented to be randomised to receive either an epidural or continuous midwifery care, also found women who had an epidural were more likely to stop breastfeeding in the first six months [4]. Although this previous study had a crossover rate too high to permit a meaningful intention-to-treat analysis, women who felt strongly one way or the other about epidurals would be unlikely to consent to randomisation and so the likely confounding effect of personal choice would be reduced.

An alternative explanation for these results is that the association between breastfeeding and intrapartum analgesia is due to the pharmacological effect of the analgesic agents. There is a growing body of evidence that the fentanyl component of epidurals may be associated with sleepy infants and difficulty establishing breastfeeding. Lower neurologic and adaptive capacity scores have been reported among infants whose mothers had a bupivacaine and fentanyl epidural compared with mothers who had bupivacaine with sufentanil or bupivacaine alone [14]. Another study found that mothers who had had intrapartum fentanyl were less likely to be breastfeeding at discharge from hospital and that there was a dose-response relationship between fentanyl and bottle feeding, independent of other factors [3]. However the strongest evidence to date comes from a recent randomised controlled trial in which 177 women who had previously breastfed were randomised to receive an epidural containing either no fentanyl, intermediate dose fentanyl (up to 150 μg fentanyl) or high dose fentanyl (>150 μg fentanyl) [15]. Women randomised to the high dose fentanyl group were more likely to have stopped breastfeeding at six weeks postpartum than women who were randomised to the other two groups [15]. In our study we did not have information about the dosage or timing of the analgesic agents used for the individual women, so cannot draw any conclusions about this. The standard concentration of epidural fentanyl solution in the ACT at the time of this study was 3.3 μg/ml. This was lower than the usual concentrations used elsewhere in Australia and New Zealand at that time, which in many centres was 5 μg/ml, although this was variable. The usual dose now is 4 μg/ml, reflecting a slight increase in fentanyl concentration in the ACT but a reduction in concentration for some centres elsewhere (Dr Cliff Peady, Acting Director Anaesthetics, The Canberra Hospital, personal communication).

## Conclusion

Whatever the underlying mechanism, women in this cohort who chose or needed epidural analgesia were more likely to partially breastfeed their infant and to experience difficulty breastfeeding in the few days after birth, and also to stop breastfeeding in the first 24 weeks postpartum. Even though this relationship may not be causal, it is important that women who are at higher risk of breastfeeding cessation are provided with adequate breastfeeding assistance and support, both in the initial postpartum period and the following few months.

## Declaration of competing interests

The author(s) declare that they have no competing interests.
